# Ferroptosis inhibition and AMPK activation: key mechanisms of soy isoflavones against cerebral injury

**DOI:** 10.3389/fimmu.2026.1663986

**Published:** 2026-04-02

**Authors:** Cailian Wu, Tiantian Luo, Jinfeng Huang, Ruikang Mo

**Affiliations:** 1 Department of Emergency, The Second Affiliated Hospital of Guangxi Medical University, Nanning, Guangxi, China; 2 Mental Health Center, The Second Affiliated Hospital of Guangxi Medical University, Nanning, Guangxi, China; 3 Department of Rehabilitation, The First People’s Hospital of Nanning, Nanning, Guangxi, China; 4 Department of Neurology, The First People’s Hospital of Nanning, Nanning, Guangxi, China

**Keywords:** AMPK signaling pathway, cerebral ischemia-reperfusion injury, ferroptosis, MCAO, soy isoflavones

## Abstract

**Background:**

Cerebral ischemia–reperfusion injury (CIRI) remains a major cause of neurological disability and lacks effective neuroprotective interventions. Soy isoflavones (SI), phytoestrogens with antioxidant and anti-inflammatory properties, have shown neuroprotective potential. Given that ferroptosis contributes to CIRI pathogenesis and AMP-activated protein kinase (AMPK) regulates redox homeostasis, iron metabolism, and lipid peroxidation, we investigated whether SI protect against CIRI by activating AMPK and inhibiting ferroptosis.

**Methods:**

Male Sprague–Dawley rats subjected to middle cerebral artery occlusion (MCAO) were pretreated with SI (120 mg/kg, gavage) for 21 days. Neurological outcomes were assessed by infarct volume (TTC), brain water content, and behavioral scoring (Longa, mNSS). Ferroptosis was evaluated via cerebral Fe²^+^/total iron levels, oxidative stress indices (MDA, GSH, SOD, ROS), and protein expression of GPX4, ACSL4/3, xCT, and ferritin (FTH/FTMT). AMPK activation (p-AMPK/AMPK) was determined by Western blotting. Mechanistic validation employed RSL3 (ferroptosis inducer) and compound C (AMPK inhibitor).

**Results:**

SI pretreatment significantly reduced infarct size, alleviated edema, and improved neurological function. SI attenuated cerebral iron accumulation, suppressed ROS and MDA production, and enhanced GSH and SOD activities. Western blot analysis revealed downregulation of ACSL4 and upregulation of GPX4, ACSL3, xCT, and ferritin, consistent with ferroptosis suppression. The protective effects of SI were abolished by RSL3, confirming ferroptosis dependence. Furthermore, MCAO suppressed AMPK activation, which was restored by SI; inhibition of AMPK with compound C intensified ferroptotic injury and negated SI’s benefits, whereas co-treatment with SI reversed these detrimental effects.

**Conclusion:**

SI confer neuroprotection against CIRI by activating AMPK signaling and inhibiting ferroptosis. These findings highlight the AMPK–ferroptosis axis as a promising therapeutic target for ischemic stroke.

## 1 Introduction

Stroke represents a major factor in persistent disability and ranks as the planet’s third most prevalent cause of death worldwide (1). The World Health Organization predicts that stroke deaths will reach 78 million by 2030 (2). Ischemic stroke accounts for nearly 85% of all stroke incidents (3). Pharmacological thrombolysis to restore cerebral blood flow is the standard treatment for ischemic stroke (4). However, thrombolytic treatment entails potential complication risks. Cerebral ischemia-reperfusion injury (CIRI), a recognized complication, frequently develops upon blood flow restoration during thrombolytic or surgical interventions (5, 6). However, we do not fully understand the pathological processes underlying CIRI.

Ferroptosis constitutes a regulated cell death modality characterized by toxic buildup of lipid peroxides and compromised function of the lipid repair enzyme GPX4 (7, 8). Previous investigations have underscored the relevance of ferroptosis within CIRI pathogenesis (9). Experimental evidence confirms heightened vulnerability to middle cerebral artery occlusion (MCAO) in animals exhibiting iron overload, while iron chelation or depletion substantially diminishes CIRI-mediated cerebral injury (6, 10). Clinical studies further reveal that increased serum iron concentrations correlate strongly with poorer clinical prognoses following acute ischemic stroke (11). Recent preclinical work additionally shows ferrostatin-1 inhibition attenuates neurological injury in MCAO mouse models (12).

Soy isoflavone (SI), a phytoestrogen biosynthesized in legumes, demonstrates estrogen-mimetic bioactivity (13). Extensive research has documented SI’s beneficial roles across multiple health domains, including cardiovascular disease prevention, anti-aging actions, and anti-tumor effects (14, 15). Furthermore, SI provides neurotrophic and cytoprotective functions by significantly decreasing neuronal apoptotic activity (16). Administration of SI enhances cerebral perfusion in CIRI rats through inflammatory response suppression, ameliorating CIRI and facilitating neurological restoration (17). Additionally, this compound confers neuroprotection against CIRI via oxidative stress attenuation and mitochondrial integrity preservation (18). Nevertheless, SI’s therapeutic potential targeting ferroptosis inhibition in CIRI treatment remains unexplored. Unlike previous studies that primarily focused on SI’s antioxidant and anti-apoptotic mechanisms, our investigation specifically targets the novel AMPK-ferroptosis axis, representing a distinct mechanistic pathway for neuroprotection.

AMPK was selected as the target pathway based on its established roles in cellular energy metabolism, iron homeostasis regulation, and antioxidant enzyme expression - all critical factors in ferroptosis regulation. This investigation employed MCAO rat models to assess SI’s efficacy against CIRI and investigate ferroptosis involvement in its therapeutic actions. Our results provide novel therapeutic perspectives for clinical CIRI management.

## 2 Materials and methods

### 2.1 Main reagent

Triphenyl tetrazolium chloride (TTC) was acquired from Solarbio (China; Cat: G3005). Beyotime supplied the hematoxylin and eosin Staining Kit (China; Cat: C0105). RSL3 was sourced from Selleck (USA; Cat: S8155). The Iron Assay Kit originated from Abcam (USA; Cat. ab83366). Solarbio provided malondialdehyde (MDA) (Cat: BC0025), glutathione (GSH) (Cat: BC1175), and superoxide dismutase (SOD) assay kits (China; Cat: BC0175). Cellular ROS detection using DCFH-DA employed kits from Cell Biolabs (USA; Cat: STA-342). Compound C was obtained from EMD Millipore (USA; Cat: 171260).

### 2.2 Drug solution preparation

SI (>80% purity) was procured from Shaanxi Sciphar Natural Products Co., Ltd. (China). SI with >80% purity was selected based on standardized pharmaceutical grade requirements for preclinical studies. This purity level ensures consistent bioactive compound content while minimizing potential confounding effects from impurities. According to the manufacturer’s certificate of analysis, the SI extract primarily consists of genistein (35-45%), daidzein (30-40%), and glycitein (8-12%), with the remaining components comprising their respective glycoside forms (genistin, daidzin, glycitin). For 12 mg/mL SI solution preparation, 14.12 mg SI powder underwent dissolution in 1 mL ultrapure water, with subsequent storage at 4°C.

### 2.3 Animals

The Ethics Center of the Second Affiliated Hospital of Guangxi Medical University approved all protocols [Approval No. 2024-KY (1124)]. Sprague-Dawley male rats (250-280g; 6 weeks old) were obtained from Beijing Weitong Lihua Experimental Animal Technology (China). Male rats were specifically selected to eliminate estrogen cycle-related confounding variables that could interfere with the phytoestrogenic effects of SI. This approach allows for clearer assessment of SI’s direct AMPK-mediated neuroprotective mechanisms independent of endogenous hormonal fluctuations. Animals had unrestricted access to food and water. Ambient conditions maintained 20–24 °C temperature and 40–60% relative humidity throughout experiments. Prior to procedures, rats underwent one week of environmental acclimatization. All experimental procedures were conducted in strict accordance with the National Institutes of Health (NIH) Guide for the Care and Use of Laboratory Animals and the ARRIVE (Animal Research: Reporting of *In Vivo* Experiments) guidelines. For all surgical procedures, rats were anesthetized with isoflurane delivered in oxygen. Anesthesia was induced with 3-4% isoflurane in an induction chamber and maintained at 1.5-2% isoflurane via a nose cone. Adequate depth of anesthesia was continuously monitored and confirmed by the absence of pedal withdrawal reflex and corneal reflex, along with a stable respiratory rate. At the designated endpoints, rats were euthanized by intraperitoneal injection of sodium pentobarbital (150 mg/kg), following the guidelines of the American Veterinary Medical Association (AVMA) for the Euthanasia of Animals. Death was confirmed by the cessation of heartbeat and respiration, followed by immediate decapitation for rapid brain extraction. Brain tissues and serum samples were then collected and stored at −80°C for subsequent analysis.

### 2.4 MCAO model establishment

We created cerebral ischemia-reperfusion injury models using established MACO protocols (19). Sprague-Dawley rats first underwent isoflurane-induced inhalation anesthesia. Animals were then positioned supinely for midline cervical incision under microscopic guidance (Leica, Germany). The right common, external, and internal carotid arteries (CCA, ECA, ICA) were meticulously isolated from surrounding tissues. A minimal arteriotomy was created in the ECA using Vanes-style Spring scissors, permitting insertion of a silicone-coated 4–0 nylon monofilament (Doccol Corporation, USA; 403756PK10Re) with a rounded tip. This filament was threaded into the ICA, advancing 8–10 mm past the bifurcation to occlude the middle cerebral artery. Successful occlusion was confirmed by observing a >80% reduction in regional cerebral blood flow using laser Doppler flowmetry (Moor Instruments, UK) positioned over the ipsilateral parietal cortex. Following 6 h occlusion, filament withdrawal reinstated perfusion. The 6-hour occlusion duration was selected based on preliminary studies to produce consistent moderate-to-severe cerebral infarction (approximately 30-40% of hemisphere) suitable for detecting neuroprotective effects while minimizing mortality. Core temperature was regulated at 37.0 ± 0.5°C intraoperatively.

### 2.5 Experimental groups

Rats were randomized into three cohorts: Sham, MCAO, and SI groups. SI group animals receiveddaily intragastric SI (120 mg/kg) for 21 preoperative days. The 120 mg/kg dose was selected based on our dose-response studies (**Supplementary Figure S1**), which demonstrated optimal neuroprotective effects with this dosage in cerebral ischemia models, while the 21-day pretreatment period was chosen to ensure adequate tissue accumulation and biological activity of SI compounds. Sham and MCAO counterparts were administered equivalent saline volumes via identical route. MCAO surgery commenced 30 min post-final dosing. Post-observation, euthanasia via sodium pentobarbital overdose preceded rapid brain extraction and serum collection, with immediate storage at −80 °C.

### 2.6 Pharmacological interventions

For ferroptosis induction experiments, RSL3 (10 mg/kg, *i.p.*) was administered 1 hour before MCAO surgery. For AMPK inhibition studies, Compound C (20 mg/kg, *i.p.*) was injected 30 minutes prior to MCAO. Control groups received equivalent volumes of vehicle (DMSO in saline, <1% final concentration).

### 2.7 Neurological assessment

All neurological assessments were performed at 24 hours after reperfusion. Rat neurological function was evaluated using Longa’s 0–4 scoring system (20): 0 indicating normal mobility; 4 representing comas with locomotion inability. Evaluators remained blinded to group assignments throughout. Cognitive impairment was additionally quantified through modified neurological severity scores (mNSS) (21). Longa’s scoring criteria were as follows: 0 = no neurological deficits; 1 = failure to extend left forepaw fully; 2 = circling to the left; 3 = falling to the left; 4 = inability to walk spontaneously with depressed consciousness. mNSS evaluates motor function, sensory function, reflex responses, and balance on a scale of 0-18, with higher scores indicating greater neurological impairment.

### 2.8 Infarct volume quantification and cerebral edema measurement

Brains were sectioned coronally at 2 mm thickness. Sections underwent 37°C TTC staining for 30 minutes, followed by 10-minute immersion-fixation in 10% paraformaldehyde. Infarct percentage was computed as: infarct rate = infarct area/total slice area × 100%. Brain water content was determined according to established methodology (22). After extraction, fresh tissue mass was recorded as wet weight (WW). Samples were then desiccated to constant mass (DW) in drying ovens. Edema percentage was calculated: [(WW - DW)/WW] × 100%.

### 2.9 Iron quantification

Ferrous and total iron concentrations were measured using the Iron Assay Kit per manufacturer specifications. Optical density at 593 nm was determined spectrophotometrically. Iron measurements were performed in triplicate using freshly prepared standard solutions (0–100 mM FeSO_4_). Instrument calibration was verified using certified reference materials before each batch analysis. Background absorbance was measured using tissue-free buffer controls.

### 2.10 MDA, GSH and SOD assessments

SOD, MDA, and GSH activities were evaluated with corresponding assay kits following kit protocols. Resultant chromogenic reactions were quantified via microplate reader absorbance measurements. All biochemical assays were conducted in triplicate with concurrent analysis of kit-provided standards and quality controls. For MDA assays, 1,1,3,3-tetraethoxypropane standards (0–40 nmol/mL) were used for calibration. GSH measurements utilized reduced glutathione standards (0–60 mM), while SOD activity was calibrated against xanthine oxidase inhibition standards. Blank controls (assay buffer without tissue) and positive controls (oxidized samples) were included in each experimental run.

### 2.11 Reactive oxygen species detection

Cellular ROS levels were detected using the xylenol orange (XO) - based ferrous ion oxidation - xylenol orange (FOX) kit per manufacturer guidelines. Absorbance signals (at a characteristic wavelength of 560 nm for the XO - Fe³^+^ complex) were measured with a UV - visible spectrophotometer for quantitative analysis. ROS detection was performed in triplicate with H_2_O_2_ - treated positive controls and PBS - treated negative controls included in each experiment. The absorbance response for ROS detection was calibrated using known concentrations of H_2_O_2_ (0–200 mM). Background absorbance was measured in cells treated with vehicle alone (to eliminate interference from non - specific absorption of the vehicle or cellular components).

### 2.12 Western blot analysis

Western blot analysis targeted key ferroptosis regulatory proteins including glutathione peroxidase 4 (GPX4), acyl-CoA synthetase long-chain family members 3 and 4 (ACSL3, ACSL4), cystine/glutamate antiporter (xCT), and ferritin subunits (FTMT, FTH), as well as AMPK signaling pathway components (AMPK, phospho-AMPK). Brain tissue proteins were isolated using RIPA buffer containing protease inhibitors (PMS). After BCA quantification, we separated proteins on 10% SDS-PAGE gels and transferred them to PVDF membranes. Membranes were blocked with skim milk, then probed with appropriately diluted primary antibodies during overnight incubation at 4 °C. After washing, horseradish peroxidase-conjugated secondary antibodies were applied for 2 hours. Chemiluminescent visualization revealed target bands using Prime Western Blotting Reagent. Band intensities were quantified via ImageJ densitometry. Antibody specifications appear in **Supplementary Table S1**.

### 2.13 Statistical analysis

SPSS 22.0 facilitated all statistical analyses. Continuous variables are presented as mean ± SD ( $\bar{\text{x}}$ ± s). Between-group comparisons employed one-way ANOVA followed by Tukey’s HSD *post-hoc* test for pairwise comparisons when significant main effects were detected. Significance threshold was established at *P* < 0.05.

## 3 Results

### 3.1 SI alleviated CIRI and improved neurological function in MCAO rats

**Figure 1:**
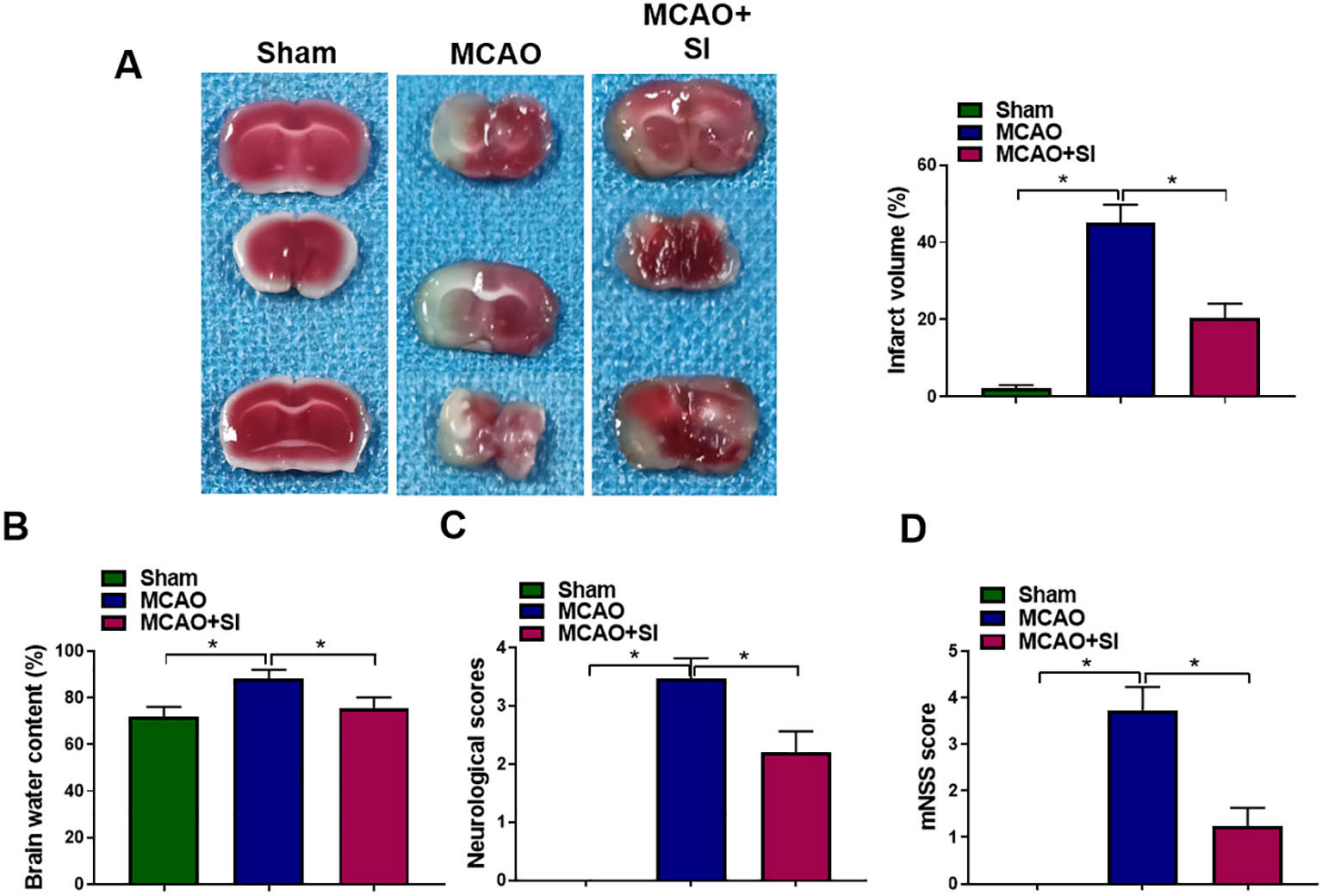
SI alleviated CIRI and improved the neurological function of MCAO rats. **(A)** The infarct area of rat brains in each group. **(B)** The water content of rat brains in each group. **(C)** The neurological deficit score of rats in each group. **(D)** The mNSS score of rats in each group. n = 3 rats per group. *P < 0.05 (one-way ANOVA).

To evaluate SI’s therapeutic potential against cerebral ischemia-reperfusion injury, middle cerebral artery occlusion models were employed. TTC staining demonstrated substantial TTC-negative regions in MCAO group brains. SI administration significantly diminished cerebral infarct volumes (**Figure 1A**). Brain edema quantification revealed markedly elevated water content in ischemic rats, substantially reduced by SI treatment (**Figure 1B**). Neurological assessment indicated higher deficit scores and mNSS values in MCAO animals versus controls. SI intervention effectively ameliorated these pathological increases (**Figures 1C, D**).

### 3.2 SI attenuated the ferroptosis in MCAO rats

**Figure 2:**
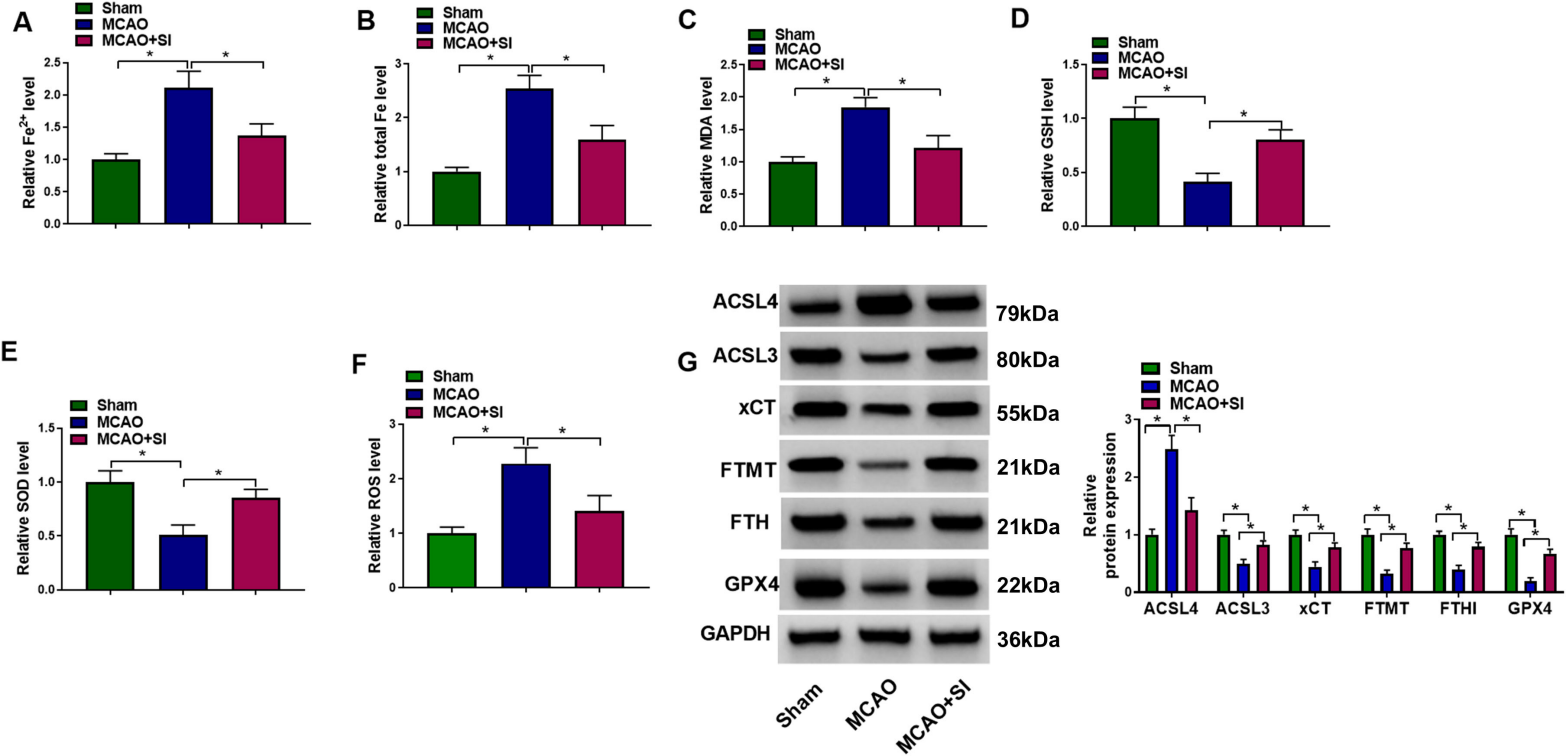
SI suppressed the ferroptosis in MCAO rats. **(A)** The total Fe levels of rat in each group. **(B)** The Fe^2+^ levels of rats in each group. **(C)** The MDA levels of rats in each group. **(D)** The GSH levels of rats in each group. **(E)** The SOD levels of rats in each group. **(F)** The ROS levels of rats in each group. **(G)** The protein levels of ACSL4, ACSL3, xCT, FTMT, FTH and GPX4 of rats in each group. n = 3 rats per group. *P < 0.05 (one-way ANOVA). Full western blot images can be found at **Supplementary Materials**.

Given ferroptosis involvement in CIRI pathogenesis, cerebral iron accumulation was quantified. MCAO rats exhibited significantly increased Fe²^+^ and total iron versus sham controls. SI treatment notably attenuated cerebral iron overload (**Figures 2A, B**). Oxidative stress analysis showed SI substantially decreased MDA while elevating GSH and SOD activities (**Figures 2C, E**). Additionally, TfR1 expression was examined to comprehensively evaluate iron homeostasis (**Supplementary Figure S2**). MCAO significantly increased TfR1 expression compared to Sham, indicating enhanced iron uptake under ischemic conditions. SI pretreatment markedly reduced TfR1 levels. Besides, SI suppressed ischemia-induced ROS generation (**Figure 2F**). The reliability of ROS detection was validated using PBS (negative control) and H_2_O_2_ (positive control) treatments, which showed expected baseline and elevated ROS levels, respectively (**Supplementary Figure S3**). Western blotting showed that SI decreased ACSL4 expression and increased GPX4, ACSL3, and xCT expression. Ferritin expression (FTMT, FTH) was also enhanced (**Figure 2G**). These results indicated AMPK’s involvement in SI-mediated iron metabolism regulation.

### 3.3 SI treated CIRI by reducing ferroptosis in MCAO rats

**Figure 3:**
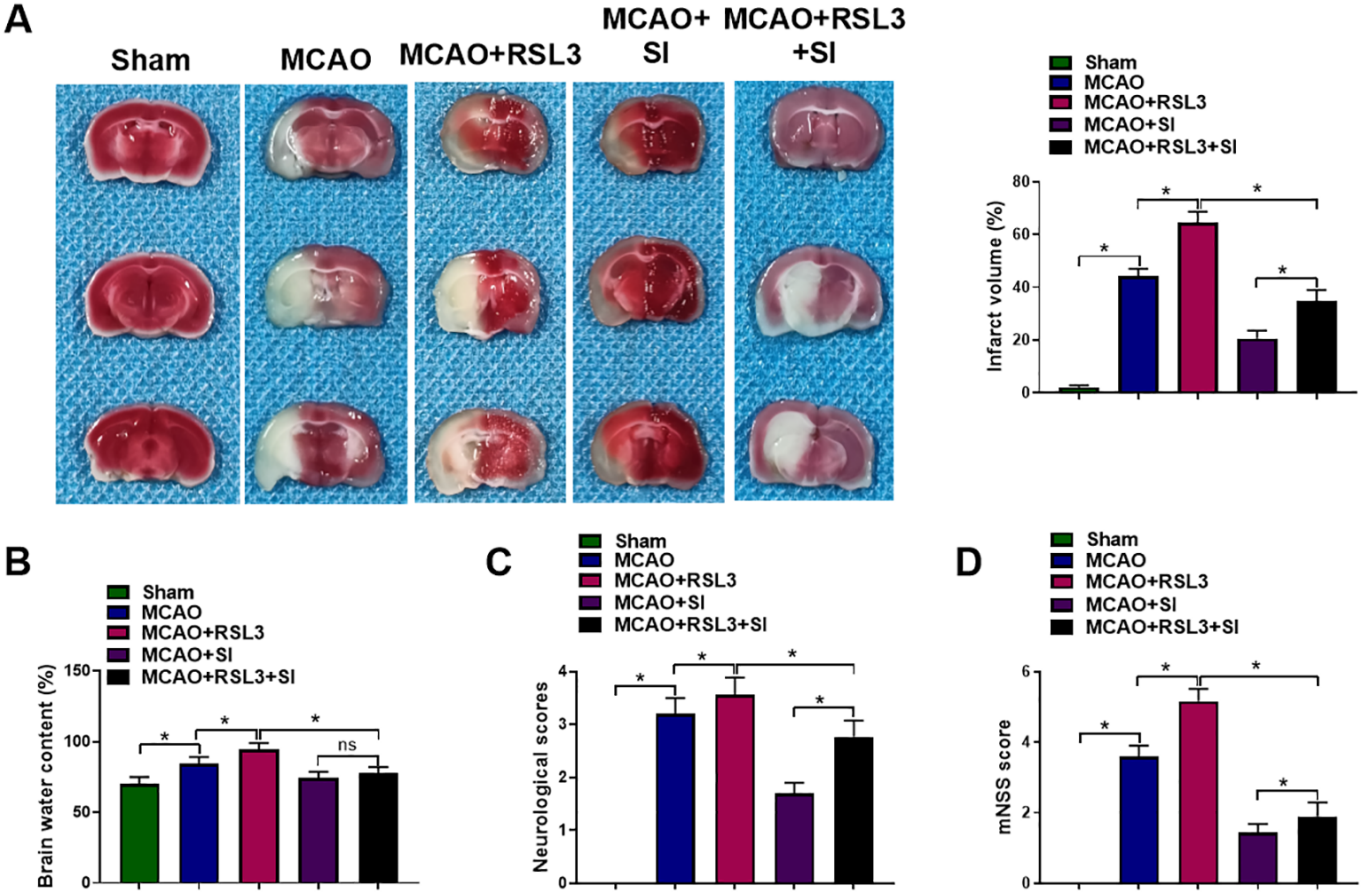
SI reduced CIRI and improved the neurological function of MCAO rats by inhibiting ferroptosis. **(A)** The infarct area of rat brains in each group. **(B)** The water content of rat brains in each group. **(C)** The neurological deficit score of rats in each group. **(D)** The mNSS score of rats in each group. n = 3 rats per group. *P < 0.05 (one-way ANOVA), ns non-significant (one-way ANOVA).

To ascertain whether ferroptosis inhibition underlies SI’s neuroprotection, the ferroptosis inducer RSL3 was co-administered. RSL3 exacerbated cerebral infarctions relative to MCAO controls (**Figure 3A**). Brain water content increased significantly in RSL3-treated animals (**Figure 3B**). Neurological impairment severity was greater in this group (**Figures 3C, D**). Critically, SI co-treatment reduced RSL3-induced exacerbation of tissue damage and functional deficits (**Figure 3**).

### 3.4 SI activated the AMPK signaling pathway in MCAO rats

**Figure 4:**
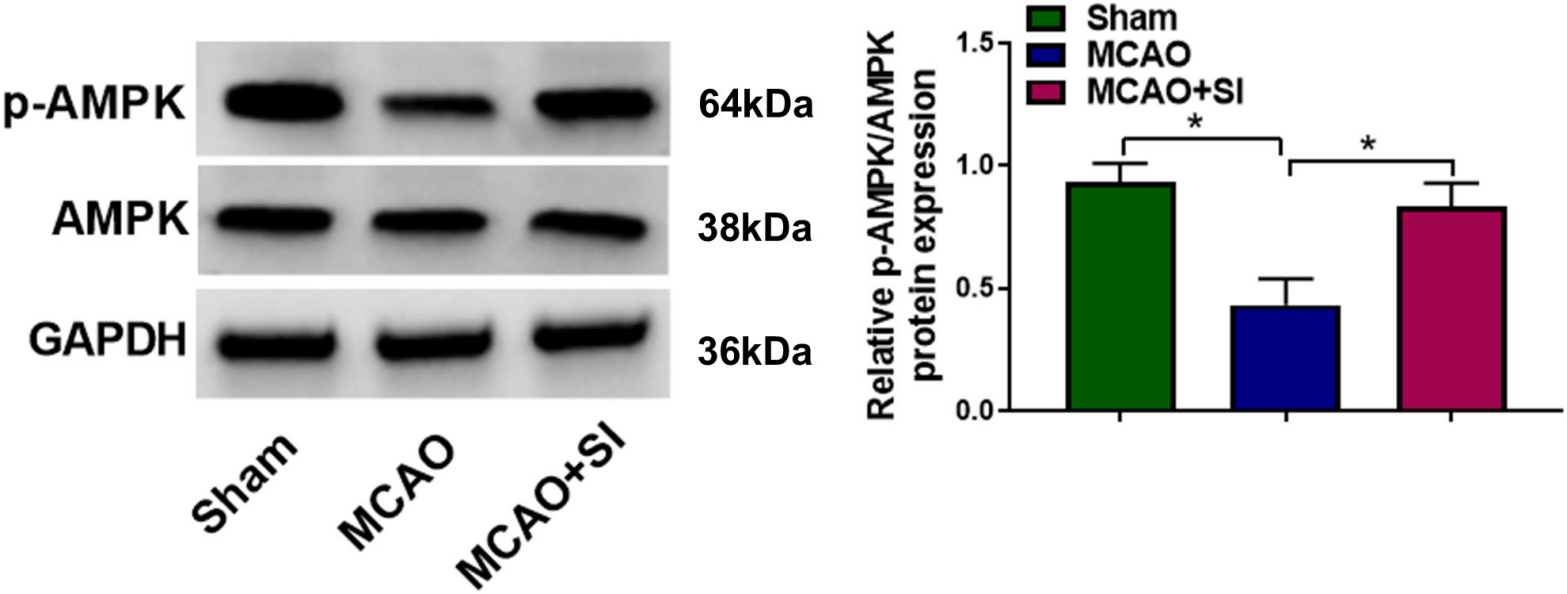
SI promoted the activation of AMPK signaling pathway in MCAO rats. n = 3 rats per group. *P < 0.05 (one-way ANOVA). Full Western blot images can be found at **Supplementary Materials**.

Subsequent investigation focused on SI’s molecular actions against CIRI. A marked reduction in the p-AMPK/AMPK ratio was observed within the brain tissue of MCAO model rats. Treatment with SI, however, substantially elevated this ratio (**Figure 4**). To further validate AMPK pathway specificity, we examined the phosphorylation status of AMPK downstream targets ACC and mTOR. As shown in **Supplementary Figure S4**, MCAO increased p-ACC and decreased p-mTOR levels, which were reversed by Compound C treatment. SI pretreatment modulated these downstream targets, and co-administration of Compound C with SI attenuated SI’s effects, confirming that SI’s protective effects are mediated through AMPK signaling.

### 3.5 SI inhibited ferroptosis via AMPK signaling pathway in MCAO rats

**Figure 5:**
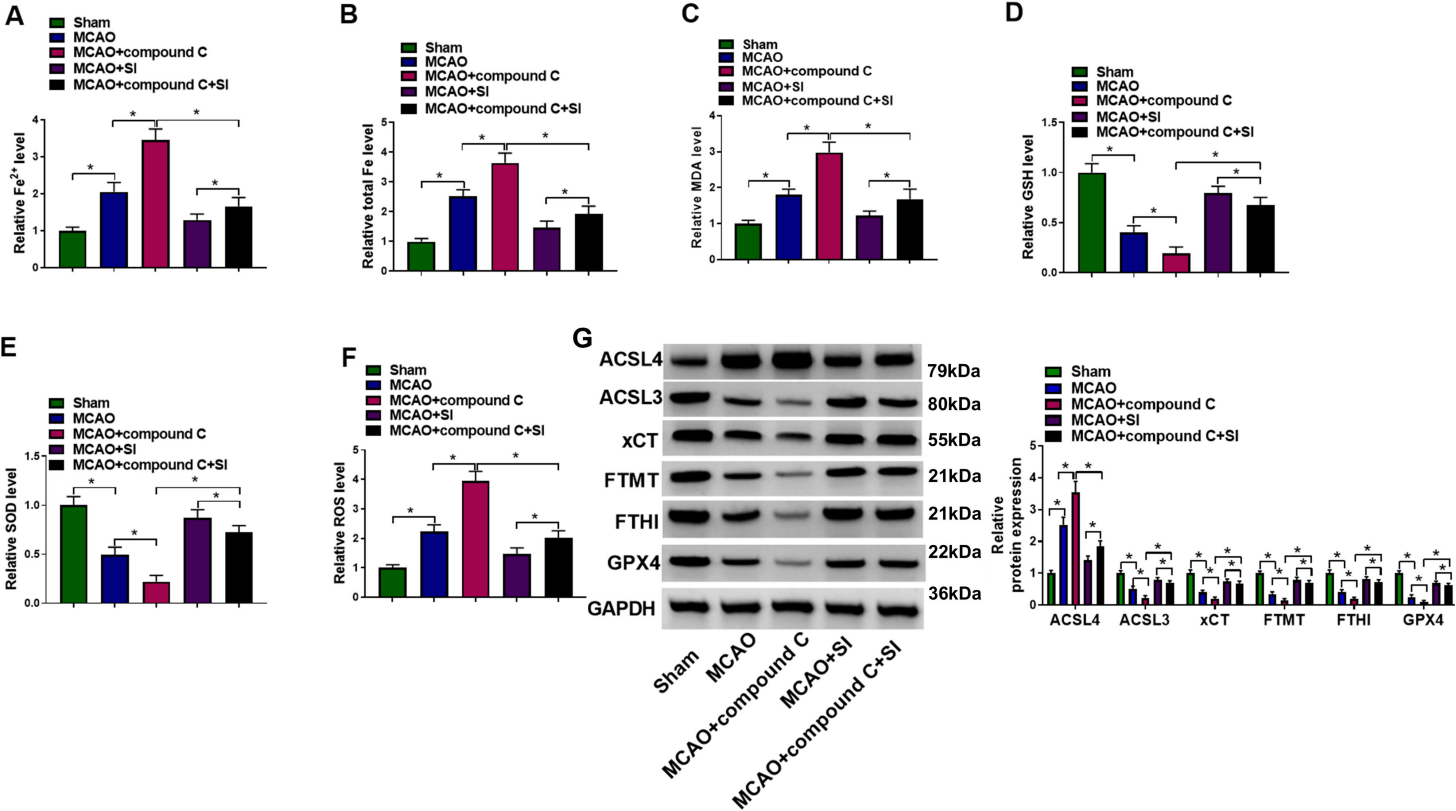
SI reduced the ferroptosis by activating in AMPK signaling pathway in MCAO rats. **(A)** The total Fe levels of rat in each group. **(B)** The Fe^2+^ levels of rats in each group. **(C)** The MDA levels of rats in each group. **(D)** The GSH levels of rats in each group. **(E)** The SOD levels of rats in each group. **(F)** The ROS levels of rats in each group. **(G)** The protein levels of ACSL4, ACSL3, xCT, FTMT, FTH and GPX4 of rats in each group. n = 3 rats per group. *P < 0.05 (one-way ANOVA). Full western blot images can be found at **Supplementary Materials**.

To define the AMPK pathway’s role, compound C was administered to inhibit AMPK. Relative to MCAO controls, compound C elevated cerebral Fe²^+^ concentrations and total iron content (**Figures 5A, B**). Compound C also significantly increased MDA levels while markedly decreasing GSH and SOD levels compared to the MCAO group (**Figures 5C-E**). Furthermore, compound C notably elevated ROS generation in rat brain tissues relative to MCAO controls (**Figure 5F**). Additionally, compound C administration significantly upregulated ACSL4 expression, whereas it downregulated expression of GPX4, ACSL3, xCT, FTH, and FTMT (**Figure 5G**). Notably, SI co-administration effectively counteracted the pro-ferroptotic consequences induced by compound C (**Figure 5**).

### 3.6 SI treated CIRI by attenuating ferroptosis via AMPK signaling pathway in MCAO rats

**Figure 6:**
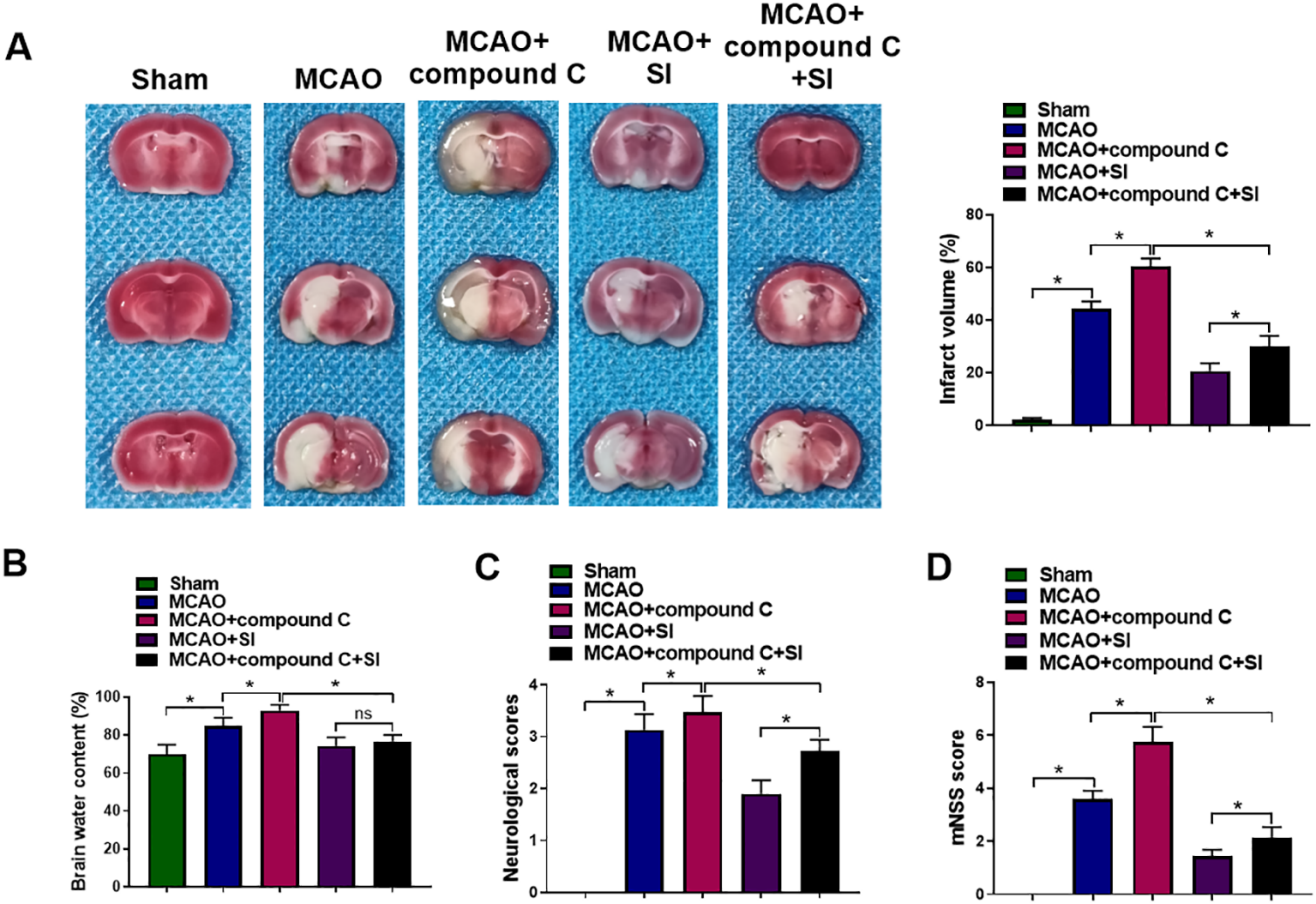
SI reduced CIRI and improved the neurological function of MCAO rats via inhibiting ferroptosis through AMPK signaling pathway. **(A)** The infarct area of rat brains in each group. **(B)** The water content of rat brains in each group. **(C)** The neurological deficit score of rats in each group. **(D)** The mNSS score of rats in each group. n = 3 rats per group. *P < 0.05 (one-way ANOVA), ns non-significant (one-way ANOVA).

The contribution of AMPK signaling to SI’s therapeutic impact on CIRI was further assessed. Administration of compound C significantly enlarged the cerebral infarct area and increased brain water content compared with the MCAO group (**Figures 6A, B**). Compound C also exacerbated neurological impairment in rats (**Figures 6C, D**). In contrast, SI treatment effectively reversed these detrimental outcomes (**Figure 6**). Collectively, these results confirm that SI alleviates CIRI by suppressing ferroptosis, an effect dependent on activation of the AMPK signaling pathway.

## 4 Discussion

This study provides initial evidence for the neuroprotective properties of SI in CIRI. Treatment with SI significantly ameliorated CIRI manifestations in MCAO model rats. Observed benefits comprised diminished cerebral infarct volume, attenuated brain edema, and improved neurological function. Further analysis indicated that SI’s protective actions involve ferroptosis inhibition, evidenced by reduced iron accumulation, lowered lipid peroxidation, and decreased reactive oxygen species. Based on mechanistic investigations, the therapeutic mechanism of SI against CIRI appears mediated by attenuation of ferroptosis through AMPK pathway activation.

Stroke pathophysiology remains incompletely understood, contributing to limited effective therapies. Rapid cerebral reperfusion represents a critical management strategy (23). Among stroke complications, CIRI is particularly consequential due to its capacity to induce substantial impairment of brain function (24). Minimizing CIRI impact is therefore a vital therapeutic objective. The MCAO paradigm serves as the predominant experimental model in ischemic stroke research (25). CIRI triggers neuronal death, precipitating cerebral infarction, edema, and cognitive dysfunction (26). In this work, MCAO rats displayed increased cerebral infarct area, elevated brain water content, and evident neurological deficits. These outcomes confirm effective establishment of the CIRI model.

Ferroptosis, a recently characterized form of regulated cell death, features iron-dependent accumulation of lethal lipid peroxides (27). Strong evidence shows that ferroptosis contributes to CIRI. Inhibiting ferroptosis can reduce CIRI damage (28). ACSL4 serves as a key determinant of cellular vulnerability to this process and represents a notable ferroptosis biomarker (29). Prior research established that ACSL4 aggravates CIRI by promoting ferroptotic death and enhancing neuroinflammation (30). Chen and colleagues demonstrated that rosiglitazone alleviates neurological deficits in CIRI mice through specific ACSL4 inhibition (31). GPX4 functions as a principal ferroptosis regulator, crucially preventing toxic lipid peroxide accumulation (32). Alim et al. reported that augmenting GPX4 activity ameliorates CIRI-induced brain injury via ferroptosis reduction (28).

SI, a soybean-derived flavonoid, displays diverse estrogen-mimetic effects. Its competitive binding to estrogen receptors and lipophilic nature facilitate blood-brain barrier penetration, enabling central nervous system protection (33). Multiple studies confirm SI’s protective actions against CIRI, involving suppression of apoptosis, oxidative stress, mitochondrial dysfunction, and inflammation (34). The potential involvement of ferroptosis inhibition in SI’s CIRI alleviation remained unexplored. Our data indicate that SI administration effectively reduced cerebral infarction volume, attenuated brain edema, and improved neurological scores in MCAO rats. Furthermore, SI treatment significantly decreased cerebral iron deposition while lowering oxidative stress markers and reactive oxygen species generation. Molecular analysis revealed SI-mediated upregulation of GPX4, ACSL3, xCT, FTMT, and FTH1 expression, alongside ACSL4 downregulation. To clarify ferroptosis’s role in SI’s therapeutic mechanism, we utilized RSL3, a specific GPX4 inhibitor critical for ferroptosis execution (35). Notably, RSL3 administration weakened the protective effect of SI against CIRI. Collectively, these results establish that SI intervention confers protection against brain injury and neurological impairment in MCAO rats by effectively inhibiting ferroptosis.

AMPK, an ancient serine/threonine kinase, functions as a cellular energy sensor (36). It shows broad tissue distribution, with particularly high abundance in the brain (37). Post-cerebral ischemia, AMPK activation stimulates Hypoxia-Inducible Factor 1-alpha (HIF-1α), elevating phosphofructokinase (PFK) expression. This cascade enhances cerebral metabolic activity, sustaining energy provision while reducing CIRI-mediated neuronal injury (38). Studies indicate AMPK activation diminishes infarct volume, improves neurological outcomes, and confers neuroprotection (39). Additionally, AMPK serves a critical role in cerebral glucose regulation. Its activation during ischemia modulates glucose utilization, suppresses post-ischemic glucose intolerance, and attenuates neuronal damage (40). Our investigation detected diminished p-AMPK/AMPK levels in MCAO rats, whereas SI administration elevated this ratio, signifying AMPK pathway stimulation by SI. To assess whether SI alleviates CIRI via AMPK activation, compound C was administered to block AMPK signaling. Inhibition of this pathway markedly intensified brain injury and ferroptotic activity, while SI treatment effectively mitigated these adverse outcomes. Notably, Compound C did not completely eliminate the protective effects of SI. Two factors may account for this observation. First, although compound C is commonly used as an AMPK inhibitor, it can also influence other cellular targets; therefore, future studies using genetic strategies such as AMPK knockdown are needed to more clearly verify AMPK-specific actions. Second, the therapeutic effects of SI on CIRI involve multiple pathways beyond ferroptosis, and further investigation of these additional mechanisms will provide more robust theoretical support for its clinical translation. The mechanistic link between AMPK activation and ferroptosis inhibition likely involves multiple pathways. AMPK activation can enhance cellular antioxidant capacity through upregulation of antioxidant enzyme expression and glutathione synthesis, directly supporting GPX4 function. Additionally, AMPK may modulate lipid metabolism by influencing ACSL4 expression and activity, thereby reducing the substrate availability for lipid peroxidation. AMPK also promotes cellular iron homeostasis through regulation of ferritin expression, limiting free iron availability for ferroptotic processes. Our study focused on AMPK phosphorylation status as the primary readout of pathway activation. Investigation of downstream targets such as mechanistic Target of Rapamycin (mTOR) and UNC-51-Like Kinase 1 (ULK1) would provide a more comprehensive understanding of the signaling cascade involved. The connection between ferroptosis and neuroinflammation is particularly relevant to CIRI pathogenesis. Ferroptosis releases damage-associated molecular patterns (DAMPs) including HMGB1 and oxidized lipids, which activate microglia and astrocytes through TLR4/NF-κB signaling, perpetuating neuroinflammatory cascades (41). AMPK activation by SI not only suppresses ferroptosis but also inhibits NF-κB-mediated pro-inflammatory cytokine production (TNF-α, IL-1β, IL-6) while promoting anti-inflammatory M2 microglial polarization (42). Furthermore, lipid peroxidation products generated during ferroptosis, particularly 4-hydroxynonenal (4-HNE), directly activate inflammatory pathways and compromise blood-brain barrier integrity (43). By inhibiting ferroptosis through AMPK activation, SI interrupts this ferroptosis-inflammation positive feedback loop, providing dual neuroprotection against both oxidative and inflammatory damage in CIRI.

Our findings hold translational relevance for CIRI management. Firstly, SI exhibits considerable promise as a therapeutic candidate. Secondly, ferroptosis constitutes a key driver of CIRI pathogenesis. Finally, pharmacological agents targeting ferroptosis inhibition and AMPK modulation may offer novel CIRI treatment approaches. Several limitations warrant acknowledgment: The sample size of n=3 per group, while standard for initial mechanistic studies, may limit statistical power for detecting subtle differences in heterogeneous stroke models. Future studies with larger cohorts are warranted to validate these findings. Absence of cellular investigations restricts detailed molecular mechanism elucidation regarding SI’s actions. Furthermore, lack of clinical validation raises translational concerns, as rodent models incompletely recapitulate human CIRI complexity. Additionally, the SI extract used contains multiple isoflavone components, and the relative contribution of individual compounds (genistein, daidzein, glycitein) to the observed neuroprotective effects remains to be determined. Future studies employing single-compound validation would help identify the primary active constituents. The clinical translatability of our findings faces several challenges, including the significant differences between rodent models and human stroke pathophysiology, as well as the practical limitations of prophylactic SI administration in clinical settings. These factors must be carefully considered in future translational studies. Subsequent research should address these constraints through expanded cohorts, deeper mechanistic exploration, and clinical correlation to enhance applicability.

## 5 Conclusion

Collectively, this work demonstrates that SI administration effectively ameliorates CIRI in rats by suppressing ferroptosis through AMPK pathway activation. These data strongly support SI’s therapeutic promise for CIRI intervention.

## Data Availability

The original contributions presented in the study are included in the article/**Supplementary Material**. Further inquiries can be directed to the corresponding author.

## Supplementary material

Supplementary Figure 1 Dose-response effects of SI on MCAO-induced cerebral infarction. Representative TTC staining of brain sections from rats pretreated with SI at 60, 120, and 240 mg/kg, demonstrating dose-dependent reduction in infarct volume (white area). The 120 mg/kg dose showed optimal neuroprotective effects.

Supplementary Figure 2 Validation of AMPK downstream targets. Western blot analysis of p-ACC/ACC and p-mTOR/mTOR ratios in brain tissues from different treatment groups. β-actin served as loading control. n = 3 rats per group. Full Western blot images are available in **Supplementary Materials**.

Supplementary Figure 3 TfR1 expression in brain tissues. Western blot analysis of transferrin receptor 1 (TfR1) protein levels across treatment groups. β-actin served as loading control. n = 3 rats per group. Full Western blot images are available in **Supplementary Materials**.

Supplementary Figure 4 ROS detection with positive and negative controls. Relative ROS levels in brain tissues, including PBS treatment (negative control) and H_2_O_2_ treatment (positive control) to validate the experimental system. n = 3 per group. * *P* < 0.05 (one-way ANOVA).
