# Supplementary figures and images for: Ferroptosis inhibition and AMPK activation: key mechanisms of soy isoflavones against cerebral injury
Source: Front Immunol. 2026 Apr 2;17:1663986. doi: 10.3389/fimmu.2026.1663986 (PMC13082995; doi:10.3389/fimmu.2026.1663986)

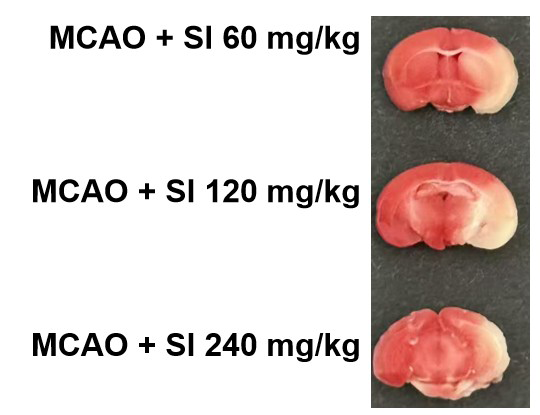

Supplement: Supplementary Figure 1 — Dose-response effects of SI on MCAO-induced cerebral infarction. Representative TTC staining of brain sections from rats pretreated with SI at 60, 120, and 240 mg/kg, demonstrating dose-dependent reduction in infarct volume (white area). The 120 mg/kg dose showed optimal neuroprotective effects. [file Image1.tif]

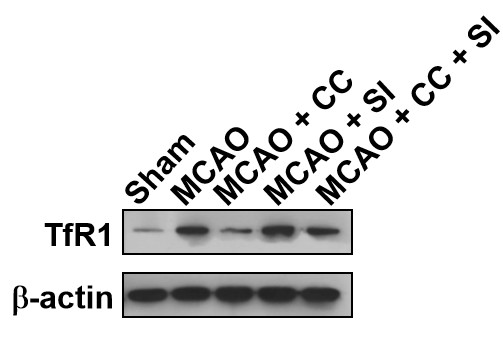

Supplement: Supplementary Figure 2 — Validation of AMPK downstream targets. Western blot analysis of p-ACC/ACC and p-mTOR/mTOR ratios in brain tissues from different treatment groups. β-actin served as loading control. n = 3 rats per group. Full Western blot images are available in Supplementary Materials. [file Image2.tif]

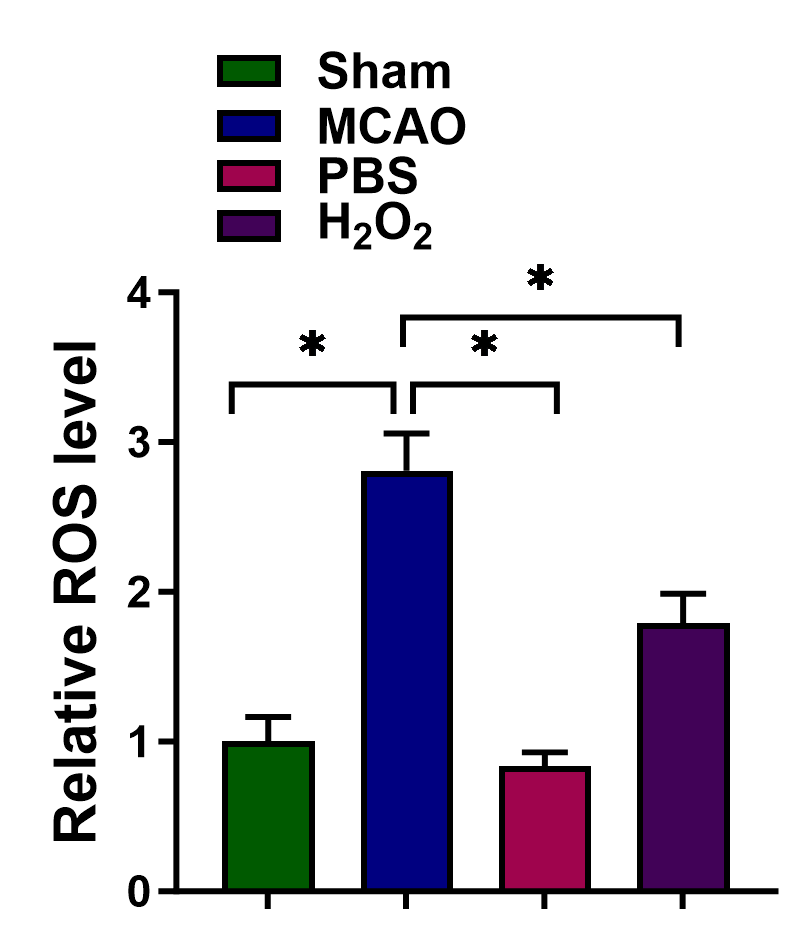

Supplement: Supplementary Figure 3 — TfR1 expression in brain tissues. Western blot analysis of transferrin receptor 1 (TfR1) protein levels across treatment groups. β-actin served as loading control. n = 3 rats per group. Full Western blot images are available in Supplementary Materials. [file Image3.tif]

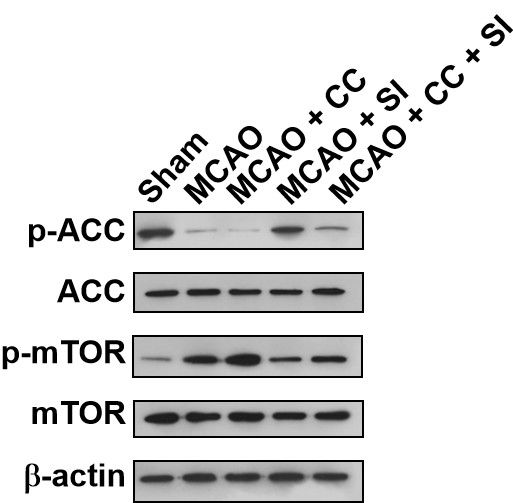

Supplement: Supplementary Figure 4 — ROS detection with positive and negative controls. Relative ROS levels in brain tissues, including PBS treatment (negative control) and H2O2 treatment (positive control) to validate the experimental system. n = 3 per group. * P < 0.05 (one-way ANOVA). [file Image4.tif]
